# Particulate Matter–Induced Health Effects: Who Is Susceptible?

**DOI:** 10.1289/ehp.1002255

**Published:** 2010-10-20

**Authors:** Jason D. Sacks, Lindsay Wichers Stanek, Thomas J. Luben, Douglas O. Johns, Barbara J. Buckley, James S. Brown, Mary Ross

**Affiliations:** National Center for Environmental Assessment, Office of Research and Development, U.S. Environmental Protection Agency, Research Triangle Park, North Carolina, USA

**Keywords:** children, genetics, lifestyle, minorities, outdoor air, particulate matter, susceptible populations

## Abstract

**Background:**

Epidemiological, controlled human exposure, and toxicological studies have demonstrated a variety of health effects in response to particulate matter (PM) exposure with some of these studies indicating that populations with certain characteristics may be disproportionately affected.

**Objective:**

To identify populations potentially at greatest risk for PM-related health effects, we evaluated epidemiological studies that examined various characteristics that may influence susceptibility, while using results from controlled human exposure and toxicological studies as supporting evidence. Additionally, we formulated a definition of susceptibility, building from the varied and inconsistent definitions of susceptibility and vulnerability used throughout the literature.

**Data synthesis:**

We evaluated recent epidemiological studies to identify characteristics of populations potentially susceptible to PM-related health effects. Additionally, we evaluated controlled human exposure and toxicological studies to provide supporting evidence. We conducted a comprehensive review of epidemiological studies that presented stratified results (e.g., < 65 vs. ≥ 65 years of age), controlled human exposure studies that examined individuals with underlying disease, and toxicological studies that used animal models of disease. We evaluated results for consistency across studies, coherence across disciplines, and biological plausibility to assess the potential for increased susceptibility to PM-related health effects in a specific population or life stage.

**Conclusions:**

We identified a diverse group of characteristics that can lead to increased risk of PM-related health effects, including life stage (i.e., children and older adults), preexisting cardiovascular or respiratory diseases, genetic polymorphisms, and low-socioeconomic status. In addition, we crafted a comprehensive definition of susceptibility that can be used to encompass all populations potentially at increased risk of adverse health effects as a consequence of exposure to an air pollutant.

To examine whether particulate matter (PM) differentially affects certain populations, epidemiological studies often conduct stratified analyses, where a greater association between PM and the health effect being examined in one subgroup compared with another provides evidence for a population that may be more susceptible to PM-related health effects. Additionally, controlled human exposure and toxicological studies can provide supporting evidence through the examination of individuals with underlying disease and animal models of disease, respectively. Often the terms “susceptible” and “vulnerable” have been used to characterize these subgroups; however, inconsistency and overlap in these definitions complicate the identification of populations that may be at greatest risk.

In this review, we integrate the evidence from recent epidemiological studies with supporting evidence from controlled human exposure and toxicological studies to identify the characteristics of populations susceptible to PM-related health effects. This review is not intended to be an exhaustive overview of the recent PM literature, but instead a comprehensive evaluation of studies that examined characteristics of potentially susceptible populations.

## Defining Susceptibility

The concept of susceptibility is derived from the interindividual variation in human responses to air pollutants, resulting in some populations being at increased risk for air-pollutant–related health effects (Kleeberger and Ohtsuka 2005). “Susceptibility” and “vulnerability” have often been used as distinct terms for identifying these populations, with “susceptibility” referring to biological or intrinsic factors (e.g., life stage, sex) and “vulnerability” referring to nonbiological or extrinsic factors [e.g., socioeconomic status (SES), differential exposure]. However, their definitions vary across reports and studies. We provide some examples below.

- American Lung Association (2001). *Susceptible:* greater likelihood of an adverse outcome given a specific exposure, compared with the general population; includes both host and environmental factors (e.g., genetics, diet, physiologic state, age, and sex, social, economic, and geographic attributes). *Vulnerable:* periods during an individual’s life when they are more susceptible to environmental exposures.
- Kleeberger and Ohtsuka (2005). *Susceptible:* intrinsic [e.g., age, sex, preexisting disease (e.g., asthma) and genetics] and extrinsic (e.g., previous exposure and nutritional status) factors.
- Pope and Dockery (2006). *Susceptible:* characteristics that contribute to increased risk of PM-related health effects (e.g., genetics, preexisting disease, age, sex, race, SES, healthcare availability, educational attainment, and housing characteristics).
- Porta (2008). *Susceptible:* vulnerability; lack of resistance to disease; the dynamic state of being more likely or liable to be harmed by a health determinant. *Vulnerable:* a position of relative disadvantage, for example, because of impaired nutrition, cognition, or social position. The extent to which a person, population, or ecosystem is unable or unlikely to respond to threats; may be used as a synonym for “susceptibility.”

In addition, the terms “at-risk population” and “sensitive population” have been used in the literature to encompass these concepts more generally.

In many instances, a characteristic that increases a population’s risk for morbidity or mortality due to exposure to an air pollutant (e.g., PM) cannot be easily categorized as solely a susceptibility or vulnerability factor due to their overlapping nature, which contributes to the complexity surrounding these concepts. Thus, we developed an all-encompassing definition for the term “susceptible population” as it relates to PM: individual- and population-level characteristics that increase the risk of PM-related health effects in a population, including, but not limited to, genetic background, birth outcomes (e.g., low birth weight, birth defects), race, sex, life stage, lifestyle (e.g., smoking status, nutrition), preexisting disease, SES (e.g., educational attainment, reduced access to health care), and characteristics that may modify exposure to PM (e.g., time spent outdoors). Rather than focusing on whether a population is susceptible or vulnerable, we focus instead on the relevant question: which individual- and population-level characteristics result in increased risk of PM-related health effects?

## Study Selection

To identify potentially susceptible populations, we focused on the collective evidence evaluated in the most recent science review of the PM National Ambient Air Quality Standards (NAAQS) [U.S. Environmental Protection Agency (EPA) 2009], building upon the evidence presented in previous PM NAAQS reviews (e.g., U.S. EPA 2004). The studies considered [see Supplemental Material, Table 1 (doi:10.1289/ehp.1002255)] examined the health effects due to short-term exposure (i.e., hours to multiple days) and long-term exposure (i.e., months to years) to either both the fine PM fraction [aerodynamic diameter ≤ 2.5 μm (PM_2.5_)] and coarse PM fraction [aerodynamic diameter between 10 and 2.5 μm (PM_10–2.5_)] or only one size fraction. Studies that focused on exposure to the thoracic PM fraction [aerodynamic diameter ≤ 10 μm (PM_10_)] are also discussed to the extent that they are informative regarding health effects related to PM_2.5_ and PM_10–2.5_ exposures.

We focused on epidemiological studies that presented stratified results (e.g., males vs. females or < 65 vs. ≥ 65 years of age) because this allowed us to compare populations exposed to similar PM concentrations within the same study design. Results from epidemiological studies provided the basis for characterizing populations potentially susceptible to PM-related health effects. We recognize that epidemiological studies that focus on only one potentially susceptible population (e.g., individuals ≥ 65 years of age or children) may provide supporting evidence on whether a population is susceptible to PM-related health effects, but we do not discuss these studies in this review because of the lack of a comparison population.

We also evaluated controlled human exposure studies that examined individuals with a preexisting disease, and toxicological studies that used animal models of disease. We used these studies to determine whether there was coherence of effects across the scientific disciplines, and to examine biological plausibility for the characteristics identified in epidemiological studies that may confer susceptibility to PM-related health effects. This approach allowed us to evaluate controlled human exposure and toxicological studies that either included or did not include a comparison population. Collectively, the results from stratified analyses in epidemiological studies along with supporting evidence from controlled human exposure and toxicological studies form the overall weight of evidence that we used to assess whether specific characteristics result in a population being susceptible to PM-related health effects.

## Life Stage

Occurrence of disease is a reflection of the interaction between host and environmental factors, which varies over time (American Lung Association 2001). Specific populations, particularly children and older adults, are identified as potentially more susceptible than the general population to PM-induced effects as a result of physiological differences.

### Children

Children exposed to comparable levels of PM are potentially more susceptible than are adults because of greater time spent outdoors, activity levels, and minute volume per unit body weight, all of which can lead to an increased PM dose per lung surface area and adverse effects on the developing lungs (U.S. EPA 2004). Recent epidemiological studies examined the association between PM and childhood respiratory effects. Collectively, evidence supports increased respiratory effects (e.g., wheeze, cough, respiratory hospital admissions) from short-term PM exposure of all size fractions in children (i.e., < 18 years) compared with adults (e.g., Host et al. 2007; Peel et al. 2005).

Toxicological studies provide support for a biologically plausible mechanism for the increased risk of respiratory effects in children. Altered lung development (i.e., structure and function) was observed in mice chronically exposed to ambient urban air during prenatal and postneonatal periods (Mauad et al. 2008). Additionally, a study demonstrated that exposure of neonatal rats to iron-soot PM resulted in reduced cellular proliferation in certain regions of the lung (e.g., Pinkerton et al. 2008). Together these studies suggest that exposure to PM during critical developmental periods may result in impaired growth of the respiratory system.

### Older adults

Older adults are generally considered a susceptible population because of the gradual decline in physiological processes over time (U.S. EPA 2006). For example, dosimetric studies show reduced clearance of PM in all regions of the respiratory tract with increasing age beyond young adulthood (U.S. EPA 2009). Older adults also represent a potentially susceptible population compared with children or younger adults because of the higher prevalence of preexisting cardiovascular and respiratory diseases, which may also confer susceptibility to PM.

Epidemiological evidence indicates increased risk of cardiovascular morbidity with short-term PM exposure in older adults. Several studies reported increased cardiovascular disease (CVD) hospital admissions among older adults compared with all ages or ages < 65 years when exposed to PM_2.5_ (e.g., Pope et al. 2008), PM_10–2.5_ (e.g., Host et al. 2007), and PM_10_ (e.g., Larrieu et al. 2007). However, some studies also revealed no evidence for increased risk of cardiovascular-related hospital admissions among older adults compared with younger ages for PM_2.5_ (e.g., Metzger et al. 2004) or PM_10_ (e.g., Zanobetti and Schwartz 2005). Studies that have examined respiratory-related effects among older adults have not consistently shown associations with PM exposure, but some have reported an increase in respiratory-related hospital admissions (e.g., Fung et al. 2005).

Although the results from the epidemiological literature are mixed regarding morbidity effects from PM exposure, the evidence from controlled human exposure and toxicological studies provides biological plausibility for PM-related cardiovascular effects in older adults. Controlled human exposure studies revealed decreased heart rate variability (HRV) in older adults with or without chronic obstructive pulmonary disease (COPD) after PM_2.5_ concentrated ambient particle (CAPs) exposure (Devlin et al. 2003; Gong et al. 2004a). Using an animal model of terminal senescence, Tankersley et al. (2008) demonstrated altered baseline autonomic tone, reductions in cardiac fractional shortening, and pulmonary vascular congestion after carbon black exposure. Additionally, arrhythmias have been observed in older, but not younger, rats exposed to PM_2.5_ CAPs (Nadziejko et al. 2004).

The continuum of effects from subclinical to cardiovascular- or respiratory-related hospitalization and ultimately death is supported by epidemiological studies showing that older adults (i.e., ≥ 75 years of age in these studies) are more susceptible to nonaccidental mortality upon short-term exposure to PM_2.5_ (e.g., Franklin et al. 2007) and PM_10_ (e.g., Zeka et al. 2006b) compared with younger ages (i.e., < 75 years of age). Similar results were observed in long-term PM_2.5_ exposure studies (e.g., Naess et al. 2007).

## Sex

Evidence is not consistent for a difference in PM-related health effects by sex. However, results from dosimetric studies demonstrate sex-related differences in the localization of particles when deposited in the respiratory tract and in the deposition rate due to differences in body size, conductive airway size, and ventilatory parameters (U.S. EPA 2004). Specifically, females have proportionally smaller airways and slightly greater airway reactivity than do males (Yunginger et al. 1992).

Relatively few epidemiological studies (i.e., reviewed in U.S. EPA 2009) have conducted sex-stratified analyses, and these results are not consistent with the findings of dosimetric studies. When examining the association between short- and long-term PM_2.5_ exposure and cause-specific mortality, existing evidence suggests slightly increased risk for females for nonaccidental mortality (Franklin et al. 2007; Ostro et al. 2006), cardiovascular-related mortality (Chen et al. 2005; Franklin et al. 2007), and lung cancer mortality (Naess et al. 2007), whereas males were at increased risk for respiratory-related mortality (Franklin et al. 2007; Naess et al. 2007). Similarly, associations between short-term exposure to PM_10–2.5_ and nonaccidental and cardiovascular mortality were stronger among females than among males (Malig and Ostro 2009). Collectively, the PM_10_ results (e.g., Chen et al. 2005; Middleton et al. 2008; Wellenius et al. 2006b; Zanobetti and Schwartz 2005; Zeka et al. 2006b) do not support the associations observed in the PM_2.5_ and PM_10–2.5_ studies. For example, slightly stronger associations between PM_10_ and cardiovascular hospital admissions were observed among males than among females (Middleton et al. 2008; Zanobetti and Schwartz 2005), and stronger associations between PM_10_ and respiratory hospital admissions (Middleton et al. 2008) and respiratory mortality (Zeka et al. 2006b) were observed among females than among males. Although human clinical studies are not typically powered to detect differences in response between males and females, one study reported significantly greater decreases in blood monocytes, basophils, and eosinophils in females than in males after controlled exposures to ultrafine (UF) elemental carbon, suggesting potential sex-related differences in subclinical responses upon PM exposure (Frampton et al. 2006).

## Race/Ethnicity

Findings from recent epidemiological studies provide evidence that suggests differential susceptibility to PM-induced health effects across races and ethnicities; however, results varied across study locations. The examination of short-term PM_2.5_ exposures and mortality in nine California counties demonstrated an increased risk of mortality for whites and Hispanics but not for blacks (Ostro et al. 2006). An additional analysis in six California counties of associations with PM_2.5_ and various PM_2.5_ components showed increased risk of mortality, specifically cardiovascular mortality, in individuals of Hispanic ethnicity compared with whites (Ostro et al. 2008). In a study in 15 California counties, Hispanics were also found to be at increased risk of cardiovascular mortality with short-term PM_10–2.5_ exposures, but not nonaccidental mortality, compared with whites (Malig and Ostro 2009). Epidemiological studies that examined health effects associated with PM_10_ exposure did not examine Hispanic ethnicity or provide clear evidence for increased risk in a specific race. For example, Zanobetti et al. (2008) found evidence for increased risk of death in other races (i.e., all races except white) compared with whites in a cohort of individuals with COPD in 34 U.S. cities. However, additional multicity studies revealed no evidence for increased risk of congestive heart failure (CHF) hospital admissions (Wellenius et al. 2006b) or cause-specific mortality (Zeka et al. 2006b) when comparing white with other races or blacks, respectively, with short-term PM_10_ exposure.

## Genetic Factors

Of recent interest is the potential for gene–environment interactions to affect the relationship between ambient air pollution and the development of health effects (Kauffmann et al. 2004). Numerous studies evaluated the effect of genetic polymorphisms on responses to air pollution exposures in both animals and humans. Functionally relevant polymorphisms in genes can result in a change in the amount or function of the protein product of that gene. Investigations of gene–environment interactions often target polymorphisms in already identified candidate susceptibility genes or in genes whose protein products are thought to be involved in the biological mechanism underlying the adverse effect of an air pollutant. Findings from these studies can provide insight into mechanisms that confer susceptibility to PM-related health effects.

Given evidence that cardiovascular and respiratory effects associated with short-term PM exposure are mediated by oxidative stress (U.S. EPA 2009), new research has focused on the glutathione *S*-transferase (GST) genes, which have common, functionally important polymorphic alleles that significantly affect antioxidant function in the lung (Schwartz et al. 2005). Individuals with genotypes that result in reduced or absent enzymatic activity are likely to have reduced antioxidant defenses and potentially increased susceptibility to inhaled oxidants and free radicals. Because most populations have a high frequency of polymorphisms in the GST genes, individuals with these polymorphisms represent a potentially large susceptible population (Gilliland et al. 2004). Studies of the Normative Aging Study cohort showed that individuals with null GST mu 1 gene (*GSTM1*) alleles had a larger decrease in HRV upon short-term PM_2.5_ exposure than did individuals with at least one functional allele (Chahine et al. 2007; Schwartz et al. 2005). Further, diabetic individuals with null compared with functional *GSTM1* alleles had larger decrements in flow-mediated dilation (FMD), suggesting alterations in endothelial function (Schneider et al. 2008). A controlled human exposure study investigated the effect of allergens and diesel exhaust (DE) particles in individuals with either null genotypes for the GST genes [*GSTM1* and the GST theta-1 gene (*GSTT1*)] or single-nucleotide polymorphisms (SNPs) in the GST pi 1 gene (*GSTP1*; i.e., codon 105 variants), which are hypothesized key regulators of the adjuvant effects of DE on allergic responses (Gilliland et al. 2004). The common *GSTP1* 105 variant (i.e., A105G) results in an amino acid change from isoleucine to valine in the GSTP1 protein and pleiotropic effects on enzymatic function (Gilliland et al. 2004). Gilliland et al. (2004) demonstrated that individuals with the *GSTM1* null or the *GSTP1* I105 wild-type genotypes were more susceptible to allergic inflammation upon exposure to allergen and DE particles than were individuals with functional *GSTM1* and *GSTP1* V105 variant. These results provide evidence of a protective effect with a *GSTP1* polymorphism.

Interactions between GST genes and PM exposure were recently considered in studies of birth outcomes. An epidemiological study examined the association between high PM_10_ exposures (i.e., PM_10_ concentrations ≥ 75th percentile of the PM_10_ distribution) during the third trimester of pregnancy and preterm delivery (Suh et al. 2008). Results showed that women with the *GSTM1* null genotype were at increased risk of preterm birth compared with women who had the functional genotype. Additionally, examination of the statistical interaction between high PM_10_ concentrations during the third trimester of pregnancy and the presence of the *GSTM1* null genotype provided evidence of a synergistic effect on the risk of preterm delivery.

Another gene involved in antioxidant responses, heme oxygenase (decycling) 1 (*HMOX1*), has been examined in a recent panel study. Chahine et al. (2007) found that HRV decreased upon short-term PM_2.5_ exposure in individuals with the long GT tandem repeat polymorphism of the *HMOX1* promoter, and not in individuals with the short repeat variant. This polymorphism is thought to decrease the inducibility of *HMOX1*, whose protein product is heme oxygenase-1, an important antioxidant enzyme (Chahine et al. 2007). Furthermore, when examining a three-way interaction, the effects of PM_2.5_ exposure on HRV were more pronounced in individuals with both the long-repeat *HMOX1* polymorphism and the null *GSTM1* genotype (Chahine et al. 2007).

Additional genes have been examined to determine if specific polymorphisms increase susceptibility to PM-related health effects. A study of the Normative Aging Study cohort focused on polymorphisms in the methylenetetrahydrofolate reductase gene (*MTHFR*) at codon C677T (i.e., CT/TT *MTHFR* genotypes) or the cytoplasmic serine hydroxymethyltransferase gene (*cSHMT*) at codon C1420T (i.e., CT/TT *cSHMT* genotypes) (Baccarelli et al. 2008). The enzymes coded by these genes are involved in folate metabolism and regulate plasma homocysteine levels, which is a risk factor for CVD. The CT/TT *MTHFR* variants are linked to reduced enzymatic activity, whereas it is unclear whether this is the case for the CT/TT *cSHMT* variants (Lim et al. 2005). Additionally, *MTHFR* and *cSHMT* were found to interact such that the effect of the *MTHFR* polymorphism on the risk of CVD varied by the *cSHMT* genotype (Lim et al 2005). Baccarelli et al. (2008) found that baseline HRV was lower in individuals with the CT/TT *MTHFR* genotypes than in individuals with the CC genotype, but they observed no relationship between HRV and *cSHMT* genotypes. However, the association between HRV and PM_2.5_ exposure was modulated by both *MTHFR* and *cSHMT*. Specifically, Baccarelli et al. (2008) observed a larger HRV reduction upon PM_2.5_ exposure in individuals with CT/TT *MTHFR* genotypes or the CC *cSHMT* genotype compared with the CC *MTHFR* genotype or CT/TT *cSHMT* genotypes. These results suggest a protective effect conferred by certain gene variants of *MTHFR* and *cSHMT* on PM-mediated alterations in HRV.

Investigations of polymorphisms of the fibrinogen genes (*FGA* and *FGB*) have also been conducted. Peters et al. (2009) examined the effect of SNPs in *FGA* and *FGB* on steady-state levels of plasma fibrinogen. Because fibrinogen has been implicated in atherothrombosis, it is thought to play a role in PM-mediated CVD. In a population of myocardial infarction (MI) survivors, an increase in plasma fibrinogen levels upon PM_10_ exposure was 8-fold higher in individuals with one homozygous minor allele genotype than in individuals homozygous for the major allele of *FGB*. Therefore, the combination of inflammatory effects and higher fibrinogen levels attributed to PM exposure in individuals with certain polymorphisms could increase the risk of PM-related cardiovascular effects (Peters et al. 2009).

Collectively, these results suggest that the presence of null alleles or specific polymorphisms in genes that mediate the antioxidant response, regulate folate metabolism, or regulate levels of fibrinogen may increase susceptibility to PM-related health effects. However, in some cases genetic polymorphisms may confer protective effects, such as those demonstrated for certain *GSTP1* variants. Thus, genetic factors can modulate the relationship between ambient PM exposure and the development of health effects by either increasing or decreasing the risk of a cardiovascular or respiratory outcome.

## Obesity

Pulmonary oxidative stress resulting from inhaled PM may lead to systemic inflammation and, subsequently, increased cardiovascular risk (Dubowsky et al. 2006). As a result, studies have recently focused on chronic inflammatory conditions, such as obesity, that may modulate PM-related health effects. From 1960 to 2004, the prevalence of overweight [body mass index (BMI) ≥ 25.0 kg/m^2^)] and obese (BMI ≥ 30.0 kg/m^2^) individuals in the United States increased from 20% to 74% and 13.3% to 32.1%, respectively (National Center for Health Statistics 2006).

Numerous studies have examined whether individuals who are overweight or obese are at increased risk of adverse health effects of PM relative to people of normal weight. Epidemiological studies reported a reduction in HRV in obese compared with nonobese subjects upon PM exposure (e.g., Schwartz et al. 2005). Additionally, studies observed higher levels of inflammatory markers in the plasma [i.e., C-reactive protein (CRP), interleukin-6 (IL-6), and white blood cell (WBC) count] (Dubowsky et al. 2006) and evidence for a larger reduction in FMD (Schneider et al. 2008) in obese than in nonobese individuals in response to short-term PM_2.5_ exposure. Studies of the Veteran’s Normative Aging and Women’s Health Initiative cohorts provided evidence for an increase in inflammatory markers and cardiovascular events, respectively, upon long-term PM exposure in individuals with BMI ≥ 25 kg/m^2^ compared with < 25 kg/m^2^ (Miller et al. 2007; Zeka et al. 2006a). However, an examination of associations between 20-year exposures to PM_10_ or PM_2.5_ and subclinical atherosclerosis in the Multi-ethnic Study of Atherosclerosis cohort provided no clear evidence for differences by BMI (i.e., > 30 kg/m^2^ vs. < 30 kg/m^2^) (Diez Roux et al. 2008). The greater response observed in obese individuals to PM exposure could be due, in part, to a higher PM dose rate in obese individuals. This has been demonstrated in overweight children, where an increase in tidal volume and resting minute ventilation was observed with higher BMI (Bennett and Zeman 2004).

## Preexisting Diseases

The National Research Council (2004) has emphasized the need to evaluate the effect of air pollution on potentially susceptible populations, including those with cardiovascular and respiratory diseases. Previous reviews of the literature suggested that preexisting cardiopulmonary diseases, as well as diabetes, may increase susceptibility to effects of PM exposure (U.S. EPA 2004). More recent epidemiological and experimental studies have built upon these conclusions to provide an additional understanding of susceptibility to PM-related health effects.

### Cardiovascular disease

Epidemiological, controlled human exposure, and toxicological studies examined whether hypertension, conditions associated with coronary artery disease [CAD; i.e., ischemic heart disease (IHD), MI, atherosclerosis], and CHF modulate PM-related health effects. Preexisting cardiovascular conditions, such as hypertension, heart disease, and coronary heart disease, are highly prevalent in the U.S. population (Table 1).

#### Hypertension

Hypertension has often been considered in stratified analyses that examine the association between short-term PM exposure and cardiovascular-related hospital admissions and emergency department (ED) visits. However, it is unclear whether preexisting hypertension modifies the associations observed. A study conducted in Utah found no evidence for increased risk of acute IHD events for PM_2.5_ exposure in individuals with preexisting hypertension compared with those without hypertension (Pope et al. 2006). This result is consistent with other studies where hypertension did not modify the association between PM and cardiovascular outcomes, such as CHF hospital admissions (e.g., Wellenius et al. 2006b). In contrast, Peel et al. (2007) found that the presence of preexisting hypertension resulted in an increased risk of ED visits for dysrhythmias and CHF with PM_10_ exposure. The potential effect of hypertension on the manifestation of PM-related cardiovascular effects is supported by a toxicological study conducted in a rat model of hypertension, which demonstrated that PM_2.5_ CAPs exposure resulted in higher mean arterial pressure compared with air controls (Sun et al. 2008). This finding suggests a relationship between PM_2.5_ exposure and hypertension that may provide biological plausibility for the worsening of hypertension-related cardiovascular outcomes observed by Peel et al. (2007).

#### CAD

We identified multiple studies that examined the effect of preexisting cardiovascular conditions associated with CAD on PM-related cardiovascular effects. In a panel study in Boston, individuals with preexisting IHD were observed to have larger alterations in HRV with PM_2.5_ exposure than did individuals without IHD (Park et al. 2005). Toxicological studies using Boston CAPs in dogs with induced myocardial ischemia, an animal model that mimics the pathophysiological effects associated with IHD, demonstrated increased ST-segment elevation and impaired myocardial blood flow in response to PM_2.5_ CAPs exposure (Bartoli et al. 2009; Wellenius et al. 2003).

Epidemiological, controlled human exposure, and toxicological studies examined the effect of previous MI on PM-induced cardiovascular effects. Wellenius et al. (2006b) found no evidence to suggest a modification of the relationship between PM_10_ and CHF hospital admissions by previous acute MI. Controlled human exposure studies investigated the effects of dilute DE or fine and UF CAPs in subjects with CAD and prior MI (Mills et al. 2007, 2008). Exposure to fine and UF CAPs, which were low in combustion-derived particles, did not result in any pronounced effects on vascular function (Mills et al. 2008). However, exposure to dilute DE promoted exercise-induced ST-segment changes, which are consistent with myocardial ischemia, and inhibited endogenous fibrinolytic capacity (Mills et al. 2007). The discrepant results in these studies may be due to medication use, because individuals with CAD (most on beta blockers) exposed to UF carbon particles had no change in HRV (Routledge et al. 2006), or due to differences in the PM. In a toxicological study using an animal model of acute MI, rats exposed to PM_2.5_ CAPs had decreased ventricular premature beats and spontaneous supraventricular ectopic beats (Wellenius et al. 2006a). In a rodent MI model of chronic heart failure, a prominent increase in the incidence of premature ventricular contraction with DE exposure was reported (Anselme et al. 2007). The discrepancy in health effects observed between toxicological studies could be due to differences in the MI model or the PM (i.e., CAPs vs. DE).

Toxicological studies also examined the effects of PM exposure in a murine model susceptible to atherosclerosis, the apolipoprotein knockout (ApoE^−/−^) mouse, which is characterized by systemic oxidative stress. ApoE^−/−^ mice acutely exposed to whole gasoline emissions resulted in electrocardiogram T-wave alterations, which were attributable to particles (Campen et al. 2006). Several studies reported relatively consistent pathophysiological effects when exposing ApoE^−/−^ mice to PM_2.5_ CAPs for several months. Chen and Nadziejko (2005) found a greater degree of atherosclerosis in ApoE^−/−^ mice than in control mice after exposure to fine CAPs (from Tuxedo, NY). Furthermore, decreased heart rate, physical activity, and temperature along with biphasic responses in HRV were observed in ApoE^−/−^ mice, but not in control mice, upon exposure to these CAPs (Chen and Hwang 2005). In addition, ApoE^−/−^ mice exposed to UF and PM_2.5_ CAPs (from Los Angeles and Tuxedo) had larger atherosclerotic lesions than those exposed to air (e.g., Araujo et al. 2008; Sun et al. 2008).

Taken together, the results from toxicological studies using models relevant to CAD provide coherence and biological plausibility for the epidemiological findings of PM-related cardiovascular effects.

#### CHF

A limited number of epidemiological studies have examined potential effect measure modification of PM-related cardiovascular effects by comparing individuals with and without preexisting CHF. In Utah, short-term PM_2.5_ exposure was associated with increased risk of hospital admissions for acute IHD events in individuals with preexisting CHF (Pope et al. 2006). Additionally, a study conducted in Cook County, Illinois, showed that individuals with preexisting CHF were at increased risk of PM-related mortality (Bateson and Schwartz 2004). However, a large multicity study revealed no evidence of increased risk of MI hospital admissions with exposure to PM_10_ in individuals with versus without CHF (Zanobetti and Schwartz 2005).

### Respiratory diseases

Epidemiological studies have examined the effect of preexisting respiratory diseases on multiple health outcomes (e.g., asthma symptoms, mortality) in response to PM exposure. In addition, animal models have been developed, and controlled human exposure studies have examined the possible effect of preexisting respiratory conditions on PM-induced health effects in an experimental setting. As was true for CVD millions of people are affected by respiratory diseases (i.e., asthma, COPD, and emphysema) in the United States, which includes approximately 9.3% of children < 18 years of age that have been diagnosed with asthma (see Table 1) (Pleis and Lucas 2009).

#### Asthma

In epidemiological studies of asthmatic children, short-term PM_2.5_ exposure was associated with an increase in medication use (Rabinovitch et al. 2006) and respiratory symptoms (i.e., cough, shortness of breath, and chest tightness) (e.g., Gent et al. 2003), and short-term PM_10_ exposure was associated with morning symptoms (Mortimer et al. 2002) and respiratory symptoms (Delfino et al. 2003). Health effects in asthmatic adults have also been demonstrated (e.g., asthma attacks with short-term PM_10_ exposure), although the evidence is more limited (Desqueyroux et al. 2002).

Toxicological studies provide coherence and biological plausibility for the findings of the epidemiological literature. In response to an acute exposure to CAPs from Detroit, an area with pediatric asthma rates three times the national average, rats with allergic airway disease exposed to PM derived from local combustion sources had eosinophil influx and increased bronchoalveolar lavage fluid protein content (Morishita et al. 2004). These findings suggest that the presence of allergic airway conditions increases susceptibility to allergic airway responses to PM_2.5_, which may be partially attributed to increased pulmonary deposition and localization of particles in the respiratory tract (Morishita et al. 2004). An additional study using rats with allergic airways disease exposed to CAPs provided evidence for increased expression of genes associated with inflammation and airway remodeling compared with nonallergic animals exposed to CAPs and allergic animals not exposed to CAPs (Heidenfelder et al. 2009). Furthermore, several toxicological studies demonstrated that PM acts as an adjuvant to enhance the severity or development of asthma (e.g., Li et al. 2009).

The results from the epidemiological and toxicological studies that focused on preexisting allergic airways disease are supported by a collection of controlled human exposure studies demonstrating that exposure to DE particles increases the allergic inflammatory response in atopic individuals (e.g., Bastain et al. 2003; Nordenhäll et al. 2001). However, not all controlled human exposure studies provided evidence for enhanced respiratory effects in asthmatic individuals. For example, a series of studies reported that healthy and asthmatic subjects exposed to CAPs of three different size fractions (PM_10–2.5_, PM_2.5_, and UF) exhibited similar respiratory responses (e.g., Gong et al. 2003, 2004b). However, these studies excluded moderate and severe asthmatics, which would be expected to show increased susceptibility to PM exposure.

#### COPD

Epidemiological panel studies that examined the effect of PM on lung function demonstrated greater declines in forced expiratory volume in 1 sec and forced vital capacity in individuals with COPD versus those without in response to PM_2.5_ exposure (e.g., Lagorio et al. 2006; Trenga et al. 2006). Conversely, in a study involving controlled human exposures to PM_2.5_ CAPs, healthy older adults experienced a somewhat greater PM-induced respiratory response (decrease in arterial oxygen saturation) than did older adults with COPD (Gong et al. 2004a). No other respiratory effects in response to PM exposure (e.g., respiratory symptoms, lung function, or airway inflammation) were observed in either group.

Dosimetric studies clearly demonstrated that COPD patients have increased dose rates of fine and UF particles and impaired mucociliary clearance relative to age-matched healthy subjects. These findings suggest that individuals with COPD are potentially at greater risk of PM-related health effects (Bennett et al. 1997; Brown et al. 2002). Support for PM-mediated exacerbation of emphysema is provided by a toxicological study using papain-treated mice. In this model, exposure to urban ambient air resulted in a PM-dependent increase in a measure of airspace enlargement (Lopes et al. 2009). The pathogenesis of emphysema is a complex process involving oxidative stress and inflammation, both of which can result from PM deposition in the respiratory tract. Collectively, these results provide preliminary evidence for biological plausibility of PM-related health effects in individuals with COPD and suggest that respiratory morbidities, excluding asthma, may also increase the susceptibility of a population to PM-related respiratory effects.

### Respiratory contributions to cardiovascular effects

Most studies that examined whether preexisting respiratory diseases increase the risk of PM-related health effects have focused on PM-induced respiratory exacerbations, but some studies have also examined whether preexisting respiratory diseases contribute to cardiovascular effects. Most epidemiological studies did not find evidence that preexisting respiratory diseases increased the risk of PM-related cardiovascular hospital admission or ED visits for a variety of cardiovascular outcomes (e.g., IHD, arrhythmias, CHF, MI); these studies examined whether preexisting respiratory infection (Wellenius et al. 2006b), pneumonia (Zanobetti and Schwartz 2005), and COPD (Peel et al. 2007) increased the risk of PM-related cardiovascular effects. However, De Leon et al. (2003) found that individuals with preexisting respiratory diseases had increased risk for PM_10_-induced circulatory mortality compared with individuals without preexisting respiratory diseases.

A controlled human exposure study demonstrated acute responses in the cardiovascular system and systemic circulation among asthmatic individuals, compared with nonasthmatics, after PM_2.5_ CAPs exposure (Gong et al. 2003). However, respiratory disease does not consistently affect cardiovascular response to PM exposure in controlled human exposure studies (e.g., Fakhri et al. 2009; Gong et al. 2004b). A toxicological study showed that the pulmonary artery lumen-to-wall ratio was decreased in an animal model of chronic bronchitis in response to PM_2.5_ CAPs, but a similar response was also observed in healthy rats (Batalha et al. 2002). Whereas the identification of characteristics of potentially susceptible populations has initially relied on epidemiological evidence, in this instance it is unclear how the epidemiological results compare with those found in the controlled human exposure and toxicological studies that focused on exposure to PM_2.5_ (e.g., CAPs). Thus, the lack of coherence across disciplines clouds whether individuals with preexisting respiratory diseases represent a population that is potentially susceptible to PM-related cardiovascular effects.

### Diabetes

Numerous studies have evaluated the potential for diabetes, a disease linked to chronic inflammation, to increase the risk of PM-related health effects. The increased interest in this population can be partially attributed to the large percentage of diabetic individuals in the United States (Table 1).

Epidemiological studies that examined whether diabetes modifies the association between cardiovascular effects and PM exposure primarily focused on short-term PM_10_ exposure. A multicity study showed > 75% greater risk of hospitalization for cardiac diseases with PM_10_ exposure among individuals with diabetes than among those without diabetes (Zanobetti and Schwartz 2002). A study conducted in Atlanta, Georgia, also showed increased risk of cardiovascular-related ED visits for PM_10_ exposure, specifically for IHD, arrhythmias, and CHF, among persons with diabetes than among those without diabetes (Peel et al. 2007). However, other studies (both multicity and single city) revealed no evidence for increased risk of cardiovascular ED visits and hospital admissions for short-term PM_2.5_ or PM_10_ exposure among persons with diabetes compared with those without diabetes (Pope et al. 2006; Wellenius et al. 2006b; Zanobetti and Schwartz 2005). Other evidence from epidemiological studies indicates that diabetes could potentially increase the risk of mortality with exposure to PM_2.5_ (Goldberg et al. 2006) and PM_10_ (Zeka et al. 2006b).

Additional epidemiological studies, as well as controlled human exposure studies, examined physiological alterations and changes in inflammatory and coagulation markers in the cardiovascular system of diabetic individuals in an attempt to provide biological plausibility for the increased risk of cardiovascular effects observed in some of the population-level studies. A panel study of individuals with diabetes demonstrated that ambient exposure to PM_2.5_ enhanced the reduction in various markers of endothelial function (Schneider et al. 2008). Liu et al. (2007) observed an increase in alterations in FMD and basal diameter upon PM_10_ exposure in persons with diabetes. On the other hand, a controlled human exposure study showed that DE elicited no prothrombotic effects in subjects with metabolic syndrome, which is characterized by alterations in physiological parameters and inflammatory markers similar to those observed in individuals with diabetes (Carlsten et al. 2008). An examination of biomarkers in individuals with diabetes who were exposed to PM revealed mixed results, including an increase in von Willebrand factor (Liao et al. 2005), an increase in thiobarbituric acid but no increases in CRP or tumor necrosis factor-α (Liu et al. 2007), and an increase in CRP and WBC count (Dubowsky et al. 2006). Although it is unclear how alterations in each of these biomarkers contribute to the potential for cardiovascular effects in individuals with diabetes, PM-induced changes in inflammation, oxidative stress, and acute-phase response may lead to more severe cardiovascular effects.

## Socioeconomic Status

In 2009, approximately 14.3% of the U.S. population was living in poverty (U.S. Census 2010). Although there are numerous indicators of SES, including economic status measured by income, social status measured by education, and work status measured by occupation, each of these linked factors can influence a population’s susceptibility to PM-related health effects (Dutton and Levine 1989). Low SES is associated with a higher prevalence of preexisting diseases, limited access to medical care, and limited access to fresh foods leading to a reduced intake of polyunsaturated fatty acids and vitamins, all of which may contribute to increased susceptibility to PM-induced health effects (Kan et al. 2008).

Indicators of SES were demonstrated in some epidemiological studies to modify health outcomes associated with PM exposure. In these studies, SES has primarily been defined at the neighborhood level (e.g., educational attainment or income within a neighborhood) to identify low, medium, and high SES areas within a study location. Educational attainment generally coincides with an individual’s income, which is correlated with other indicators of SES, such as residential environment (Jerrett et al. 2004). Epidemiological studies reported increased risk of mortality for short-term exposure to PM_2.5_ and PM_2.5_ components in low-SES groups (i.e., examined by median household income) (Franklin et al. 2008), whereas other analyses demonstrated consistent trends of increased mortality associations with PM_2.5_, PM_2.5_ species, and PM_10–2.5_ for low educational attainment groups (i.e., ≥ high school vs. < high school education) (Ostro et al. 2006, 2008; Zeka et al. 2006b). In the American Cancer Society cohort, increased lung cancer mortality with long-term PM_2.5_ exposure was observed among the subgroup with a high school education or less compared with groups with more than a high school education (Krewski et al. 2009). However, when examining PM_2.5_-related IHD mortality by education level, the reverse relationship was observed (Krewski et al. 2009).

Epidemiological studies also examined other indicators of SES, such as residential location and nutritional status, to identify their influence on the PM–health effect association. An examination of the potential modification of acute mortality effects due to PM exposure by residential location in Hamilton, Canada, using educational attainment as an indicator for SES revealed that the areas of the city with the highest SES displayed no evidence of effect measure modification, whereas the areas with the lowest SES had the largest mortality risks (Jerrett et al. 2004). Likewise, a study conducted in Phoenix used educational attainment (i.e., percentage of population with less than a high school diploma) and income (i.e., percentage of population with income below the poverty level) to represent SES (Wilson et al. 2007); the area with the lowest SES had the strongest association between PM_2.5_ and cardiovascular mortality, but the association differed when examining PM_10–2.5_, with the strongest association being observed for the area with higher educational attainment and income.

Another consequence of low SES may be decreased access to fresh foods. The effect of nutritional deficiencies was examined in a study of individuals with polymorphisms in genes associated with increased risk of CVD (Baccarelli et al. 2008). Individuals who had these genetic polymorphisms and who increased their intake (above median levels) of B_6_, B_12_, or methionine did not have alterations in HRV in response to PM_2.5_ exposure, in contrast to those individuals who did not increase nutrient intake (Baccarelli et al. 2008).

## Conclusion

Epidemiological studies have examined characteristics of populations that may render them more susceptible to PM-related health effects by conducting stratified analyses. By also considering experimental studies that examined individuals with an underlying health condition or used animal models of disease, it is possible to more thoroughly evaluate characteristics that may lead to increased susceptibility. The collective evidence across disciplines indicates that some characteristics, including life stage, genetic polymorphisms, preexisting cardiovascular and respiratory diseases, and SES, may increase the susceptibility of populations to PM-related health effects (Table 2). Additional characteristics (e.g., obesity and diabetes) were also identified.

A limitation of this review, as described throughout, is the inability to clearly state the overall strength of the evidence for some characteristics of potentially susceptible populations because of inconsistency in the evidence across epidemiological studies or lack of information from experimental studies regarding biologically plausible mechanisms. It has been noted, specifically in a recent review involving controlled human exposures to PM among potentially susceptible groups, that the relative lack of evidence of increased susceptibility may be due to a host of factors, such as medication use of the volunteers, subject selection bias, and nonspecificity of study end points, and not necessarily because these individuals did not represent populations susceptible to PM-related health effects (Huang and Ghio 2009). As a result, the collective evidence discussed within this review may not clearly identify all the characteristics of populations susceptible to PM-related health effects.

To assist in the identification of populations at increased risk for PM-related health effects, a consistent definition of susceptibility is needed. The ambiguity in the use of terms, including “susceptibility,” “vulnerability,” and “sensitivity,” across studies has to an extent increased the difficulty in focusing on the populations that have a greater likelihood of experiencing PM-related health effects. In the future, an approach similar to the one used in this review may allow the scientific community to focus on identifying the populations at increased risk to an air pollutant, regardless of their classification (e.g., susceptible, vulnerable, sensitive).

Overall, the epidemiological studies evaluated in this review, with supporting evidence from controlled human exposure and toxicological studies, identified characteristics of populations that may lead to increased susceptibility to PM-related health effects. This includes life stage, specifically children and older adults; preexisting cardiovascular (i.e., CAD) and respiratory (i.e., asthma) diseases; genetic polymorphisms; and low SES, as measured by educational attainment and income. Additionally, more limited evidence suggests an increase in PM-related health effects in individuals with diabetes, COPD, and increased BMI. Although not clearly established, the evidence evaluated also indicated potentially increased risk of PM-related health effects by sex and race/ethnicity, but these associations were not consistent across PM size fractions, health effects, and in some cases study locations. Overall, additional research is warranted to more accurately identify the characteristics of potentially susceptible populations and the biologically plausible mechanisms that result in one population being more susceptible than another to PM-related health effects. In addition, future research may enable the identification of specific PM size fractions, sources, or components that render a population more susceptible.

## Figures and Tables

**Table 1:** Percentages of the U.S. population with CVD, respiratory diseases, and diabetes.

	Age (years)									
	Adults (≥ 18)						Region			
Chronic condition/disease	*n* (× 10^6^)	(%)	18–44	45–64	65–74	≥ 75	NE	MW	S	W
CVD										

All heart disease^a^	25.1	11.2	4.1	12.2	27.1	35.8	10.6	12.3	11.3	10.2
Coronary heart disease^b^	13.7	6.1	0.9	6.7	18.6	23.6	5.3	6.7	6.4	5.5
Hypertension	51.6	23.2	8.2	32.1	50.9	57.4	21.3	23.4	25.1	21.0
Stroke	5.4	2.4	0.3	2.8	6.3	10.6	2.2	2.3	2.7	2.2

Respiratory diseases										

Asthma^c^	24.2	11.0	11.5	10.5	11.7	9.3	11.7	11.5	10.5	10.8
COPD										
Chronic bronchitis	7.6	3.4	2.3	4.2	5.5	4.8	2.8	3.2	4.0	2.9
Emphysema	3.7	1.6	0.2	2.3	4.5	5.2	1.1	1.8	1.8	1.6

Diabetes	17.2	7.7	2.2	10.6	19.9	17.2	6.3	7.7	8.3	7.6

Abbreviations: NE, Northeast; MW, Midwest; S, South; W, West. All data are from the Centers for thea Disease Control and Prevention (2008a, 2008b).

a Heart disease includes coronary heart disease, angina pectoris, heart attack, or any other heart condition or disease.

b Coronary heart disease includes coronary heart disease, angina pectoris, or heart attack.

c Prevalence data are based on adults responding to “ever told had asthma.”

**Table 2:** Susceptibility characteristics.

Characteristic	Susceptible population
Life stage	Children (< 18 years of age) Older adults (≥ 65 years of age)
Sex	—a
Race/ethnicity	—a
Genetic factors	Genetic polymorphisms: GST genes, *HMOX1*,^b^*MTHFR*,^b^*cSHMT*,^b^*FGB*^b^
Preexisting diseases	CVD: CAD Respiratory diseases: asthma, COPD^b^ Diabetes^b^
Obesity	Increased BMI^b^
SES	Low educational attainment Low income

a Of the studies evaluated, current evidence does not indicate one population is more susceptible to PM-related health effects than another.

b Additional evidence is needed to confirm whether the characteristic evaluated results in increased susceptibility to PM-related health effects.
